# Deep-Learning-Based Dose Predictor for Glioblastoma–Assessing the Sensitivity and Robustness for Dose Awareness in Contouring

**DOI:** 10.3390/cancers15174226

**Published:** 2023-08-23

**Authors:** Robert Poel, Amith J. Kamath, Jonas Willmann, Nicolaus Andratschke, Ekin Ermiş, Daniel M. Aebersold, Peter Manser, Mauricio Reyes

**Affiliations:** 1 Department of Radiation Oncology, Inselspital, Bern University Hospital, University of Bern, CH-3010 Bern, Switzerland; 2 ARTORG Center for Biomedical Research, University of Bern, CH-3010 Bern, Switzerland; 3 Department of Radiation Oncology, University Hospital Zurich, University of Zurich, CH-8091 Zurich, Switzerland; 4 Division of Medical Radiation Physics, Inselspital, Bern University Hospital, University of Bern, CH-3010 Bern, Switzerland

**Keywords:** radiotherapy, dose prediction, deep learning, quality assurance, VMAT, glioblastoma

## Abstract

**Simple Summary:**

For accurate radiotherapy, a clear definition of the geometric extent of organs and tumor volumes is important. Due to the laborious task of manually drawing contours to define these, automatic segmentation models are becoming increasingly available. These models, however, need to be visually evaluated by radiation oncology experts. This evaluation itself takes up valuable time, therefore making an efficient and clinically relevant validation of auto-segmented results desirable. An accurate 3D dose prediction model can help create dose awareness prior to the actual dose-planning step. It can provide useful information for the quality assurance of the contouring step. In this study, we trained a 3D dose predictor for volumetric modulated arc therapy (VMAT) treatment of glioblastoma patients based on an existing architecture called a cascaded 3D U-Net. We further tested this model’s sensitivity and robustness for the purpose of estimating dose changes due to contour variations.

**Abstract:**

External beam radiation therapy requires a sophisticated and laborious planning procedure. To improve the efficiency and quality of this procedure, machine-learning models that predict these dose distributions were introduced. The most recent dose prediction models are based on deep-learning architectures called 3D U-Nets that give good approximations of the dose in 3D almost instantly. Our purpose was to train such a 3D dose prediction model for glioblastoma VMAT treatment and test its robustness and sensitivity for the purpose of quality assurance of automatic contouring. From a cohort of 125 glioblastoma (GBM) patients, VMAT plans were created according to a clinical protocol. The initial model was trained on a cascaded 3D U-Net. A total of 60 cases were used for training, 15 for validation and 20 for testing. The prediction model was tested for sensitivity to dose changes when subject to realistic contour variations. Additionally, the model was tested for robustness by exposing it to a worst-case test set containing out-of-distribution cases. The initially trained prediction model had a dose score of 0.94 Gy and a mean DVH (dose volume histograms) score for all structures of 1.95 Gy. In terms of sensitivity, the model was able to predict the dose changes that occurred due to the contour variations with a mean error of 1.38 Gy. We obtained a 3D VMAT dose prediction model for GBM with limited data, providing good sensitivity to realistic contour variations. We tested and improved the model’s robustness by targeted updates to the training set, making it a useful technique for introducing dose awareness in the contouring evaluation and quality assurance process.

## 1. Introduction

Many cancers are currently treated by a combination of local and systemic therapy. Local therapy often consists of a combination of surgery and radiotherapy. The latter requires a sophisticated planning process to guarantee successful treatment. To improve the efficiency and quality of treatment planning in radiotherapy, methods for predicting possible dose distributions or dose volume histogram (DVH) curves have been introduced in previous years [1,2,3,4,5,6,7]. Since 2012, knowledge-based methods have been used to predict what is achievable in treatment planning. This was not only used for quality assurance in treatment planning [8,9,10], but also to speed up the planning process by initializing the treatment plan based on the prediction [11]. By reducing the number of inputs from the user, treatment planning becomes much more consistent, more resource-efficient, and potentially beneficial for treatment quality.

In recent years, three-dimensional dose prediction through neural networks has been shown to be a viable method for this purpose. In 2016, the first study using neural networks for dose predictions was published by Shiraishi et al. [12]. In the following years, approximately 30 more studies that tried to predict the 3D dose distribution with deep learning were published. Most of these were based on treatments with a relatively standard target orientation, with minor anatomical variations from patient to patient, such as prostate and oro/nasopharyngeal cancers [13,14,15,16]. However, there are also promising results for models in the brain, breast, and lungs [1,3,17]. In 2020, the Open-Access Knowledge-Based Planning (OpenKBP) Challenge was organized, providing an open-access dataset of head and neck treatment plans to train prediction models and evaluate them on a set of standardized metrics [18]. A total of 195 participants competed in this challenge, where the best-ranked team scored a mean absolute error (MAE) of 2.43 and 1.48 for the dose and DVH scores, respectively (see Section 2.6). Their methodology is publicly available and described as a technical note [19].

Dose prediction models are mainly used for treatment planning. This means that, in practice, in addition to the dose prediction, a second model is required to convert the predicted dose into an actual plan that is executable for the specific treatment technique. In this latter step, a final optimization incorporates individual case properties, physical constraints, and dose delivery hardware [20]. In our case, we want to use the dose prediction model for another purpose, namely contour evaluation.

The contouring of targets and organs at risk (OARs), the step that takes place prior to planning, is also subject to automation to improve efficiency and consistency with respect to the current manual process. With the implementation of artificial intelligence (AI), it is important to have specific tools for quality assurance [21]. To ensure quality, an assessment of the contours is required. Usually, visual inspection is the go-to method; however, this is a time-consuming task. For each target and OAR, every image slice needs to be visually inspected. If necessary, manual adjustments need to be made if a contour is deemed incorrect. Especially for deep-learning-based auto-segmentation models, a lack of robustness could result in unpredictable errors that can happen anywhere within the image volume [22]. There are, thus, good reasons to automate this QA step as well. There have been several attempts to provide an automatic assessment of automatic segmentations. Most of these were based on geometrical prior knowledge [23,24,25], but they lacked certainty for proper QA [26]. Moreover, more recent deep-learning-based approaches based on uncertainty maps seem to have issues [27]. To complement the current work performed on QA for auto-segmentation, we postulate that it would be beneficial to know the possible clinical impact of a specific segmentation. A deep-learning model that can give an accurate prediction of the dose received by an OAR instantly could provide the required information to assess the clinical impact of contour variations. Such dose awareness, for the evaluation of contours, can improve the efficiency of the process. Moreover, it provides clinically relevant feedback to the evaluator, who can then focus on potential segmentation errors that will have a larger impact on the treatment.

To assess the feasibility of a near-instant dose prediction model in providing dose awareness for the evaluation of auto-segmented contours, we made use of a deep-learning model to predict the dose for glioblastoma cases. Based on the network of Liu et al. [19] that was used in the OpenKBP challenge, a model was trained on a set of curated glioblastoma (GBM) cases. Unlike with current dose prediction algorithms, we wanted to verify the model’s performance for contouring quality assessment (QA). This means that, beyond specific accuracy and sensitivity, robust predictions for a broad range of situations are required. To do so, we tested our trained model on specific sets of contour alterations to assess its sensitivity. Furthermore, we stress-tested the model by using a specific worst-case test set, including rare cases where we expected it to fail. This enabled us to determine the robustness of the model and understand where further improvements are required. Subsequently, based on the outcome observed on the worst-case test set, we improved the robustness of the model by augmenting the training set with synthetically generated cases characterizing the observed failure patterns.

## 2. Materials and Methods

### 2.1. Data Collection and Preparation

Imaging data from a cohort of 125 GBM patients treated with radiotherapy at the Inselspital University Hospital (Bern, Switzerland) were available. For all patients, the planning target volume (PTV) and the OARs (organs at risk) were curated by a mutual agreement between three radiation oncology professionals. A plan was constructed using a strict dose prescription and standard templates for planning setup and dose optimization initiation for all cases. Of the first 95 cases, 60 were selected for model training, 15 were chosen as validation, and 20 were used as a test set. Of the remaining 30 cases, 10 were used to construct a worst-case test set manually, and the other 20 for improving the training by adding specific out-of-distribution cases. Section 2.5 further details how the worst-case test set and the out-of-distribution cases were designed.

### 2.2. Dose Planning

All cases were planned according to the clinical dose prescription of 60 Gy in 30 fractions based on the ESTRO-EANO guidelines [28] in the Eclipse treatment planning system (TPS) V15.06.05 (Varian Medical Systems, Palo Alto). All OARs were subject to a dose constraint, which, according to a priority list, could or could not be compromised (Table 1). All plans used a volumetric arc technique (VMAT) with a double full co-planar arc with 6 MV beams containing a flattening filter. The plans were optimized with the photon optimizer, and doses were calculated with the Anisotropic Analytical Algorithm [29]. After dose calculation, the dose was normalized so that 50% of the PTV was covered by 100% of the prescribed dose, according to the institutional clinical guidelines.

### 2.3. Training

**Figure 1:**
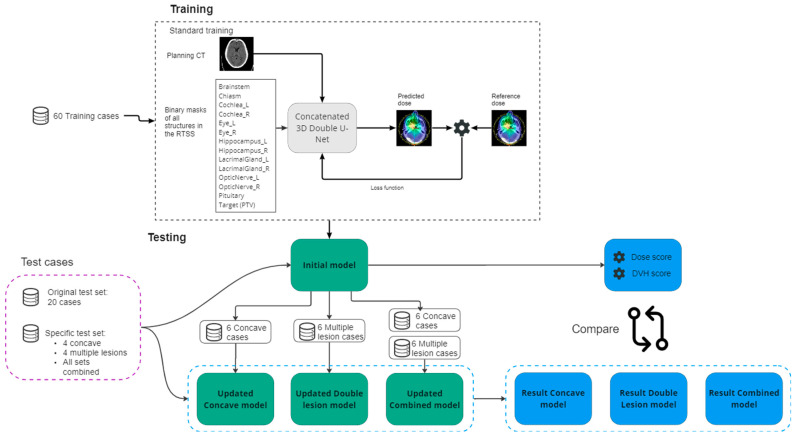
Schematic overview of the training and testing process. The upper block represents the training procedure of the initial model with its inputs and outputs. The initial model is tested on the test cases, resulting in dose and DVH scores for each test set. The initial model (green block) is updated threefold with concave cases, multiple lesion cases, and a combination of the two. The updated models are tested on the same test sets. The results are then compared (blue blocks).

The planning CT and the structures were available in DICOM format for each case. All data were converted from DICOM to NIfTI files, using the PyRaDise package [30]. The RTSS files containing the PTV and the OARs were divided into 14 separate 3D binary masks, each containing a single structure. The input files consisted of 16 3D volumes per case: the planning CT, the dose distribution, the PTV binary mask, and 13 OAR binary masks (Figure 1).

We trained a two-level cascaded 3D (C3D) U-Net [31] as the dose prediction network (i.e., the input to the second U-Net is the output of the first, concatenated with the input to the first U-Net). The U-Net is the most commonly used deep-learning neural network for dose prediction. It was first used in this context as a 2D network by Nguyen et al. [32]. Since then, many advances have been made, notably an extension to a 3D U-Net [33]. The C3D model was proposed to incorporate both global and local anatomical features for dose prediction and showed the best results among all the competing model architectures in the OpenKBP challenge [18,31].

The model input was a normalized CT volume and binary segmentation masks for each of the 13 OARs and target volume. As output, the model predicted a continuous-valued 3D dose (upscaled from [0, 1] to [0, 70 Gy] to normalize to the full range of the dose within the cohort) of the same dimension as the input. The loss was computed as follows:

Loss=0.5∗L1(reference,A)+L1 (reference,B)

where *A* and *B* are the outputs of the first and second U-Nets, respectively. In this equation, *reference* indicates the reference dose, and *L*1 refers to the *L*1 loss. All volumes were resampled to 128^3^ voxels due to GPU memory constraints. The hyperparameters for training the C3D model were unchanged from the original implementation [9], except that the number of input binary masks was updated to 14 to match the number of structures in our dataset. The model’s weights were randomly initialized using the “He” method [34]. The training process ran for 80,000 iterations, and the model with the best validation dose score was saved. All experiments were run with PyTorch1.12 on an NVIDIA RTX A5000 graphics processing unit (GPU). We trained the model five times with the same hyperparameter set but a different random seed initialization to ensure reliable convergence. Each training run took 24 h. A single inference to run the dose prediction model takes about 1–2 min on a standard consumer level PC and about 15 s with a GPU.

### 2.4. Assessing the Model’s Sensitivity

One of the goals of the dose predictor is to provide realistic dosimetry information based on the input contours. It should additionally be able to predict realistic dose changes produced by small and realistic changes to the contours of an organ (i.e., inter-expert variability [35]). To analyze the sensitivity of the dose prediction model to these changes, a specific case was chosen where the GBM target is near the left optic nerve (ONL). In practice, the optic nerves are prone to variability in contours due to the intra- and inter-fractional movement of the eyes which also affect the optic nerves. In this case, small changes in the contour of the ONL would lead to significant dose changes. To simulate this situation, ten alternative contours of the ONL were manually drawn. The dose was re-optimized and recalculated on the TPS for each alternative contour in order to serve as a reference dose. The reference doses were then compared qualitatively and quantitatively to the doses predicted by the model. The dice similarity coefficients (DSCs) for the alternatives were calculated to correlate the dose differences to the geometric discrepancy of the ONL contours.

### 2.5. Improving the Model–Worst-Case Test Set

To assess the robustness of the model, a worst-case test set was selected. Based on the analysis and evaluation of the dose score results on the standard test set and the statistical analysis of the normal distribution of the training set, a number of test cases were defined where we expected the model to fail or have difficulties. The PTVs of these cases were manually manipulated to simulate rare cases not described by the training dataset (out-of-distribution cases), as well as to present a challenge in terms of the physical limitations of obtaining perfect dose conformity. Among these 10 cases, we included (i) targets of larger and smaller size than those present in the training set; (ii) targets consisting of multiple lesions; (iii) irregular shapes, such as elongated or concave targets; and (iv) targets that present an overlap with the OARs.

According to the worst-case test set results, we gained insight into which situations the model performs poorly in and where it could benefit from additional training. Our observations showed that the prediction model struggled mostly with the physical limitations of conforming the dose according to the targets outlined for specific shapes. Where conformity of the actual dose prediction decreases with concave shapes or multiple targets close to each other, the dose predictor overestimates the dose fall-off in these regions. To increase the overall robustness of the model while also improving the model for these situations, we updated the trained model by including a set of concave-shaped target cases and a set of cases where the target consists of multiple lesions.

**Figure 2:**
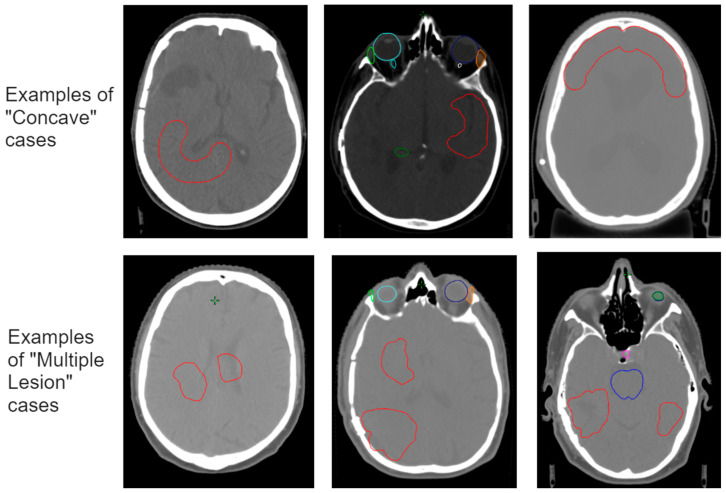
Examples of the additional training cases for the concave targets (**above**) and the multiple targets (**below**). The targets are drawn manually in red and do not represent actual tumor situations. The structures in other colors represent OARs.

The respective new training sets were constructed by manually adjusting the target volume (Figure 2). The ten cases used for both sets are from different patients and were not used in any previous model training. All new cases included dose planning according to the same protocol described above in order to serve as the reference dose.

First, we trained an updated “Concave Model” with 60 standard cases + 6 concave cases. The remaining 4 concave cases were used as the test set. Second, we trained an updated “Multiple Lesion Model” with the 60 standard cases + 6 multiple lesion cases. Again, 4 cases were used as a test set. Finally, we retrained the initial model with 60 standard cases + 6 concave cases + 6 multiple lesion cases. We tested these models on the standard test set, as well as these specific additional test cases, and compared this with the results of the initial model. An overview of the experimental setup is given in Figure 1.

### 2.6. Evaluation

The trained models were evaluated on the test set, and the prediction of the dose was compared to the actual planned dose by means of the standardized metrics used by the OpenKBP challenge [18]: the dose score and the DVH score. The dose score measures the mean error over all the voxels between the two 3D volumes. In this case, we used the whole brain to measure the dose score instead of the whole CT volume or whole body volume. Taking a larger volume dilutes the results to a more positive outcome. The DVH score is the mean error over a set of criteria specific to the given volume. For OARs, these criteria are the mean dose (Dmean) and the maximum dose to 0.1cc (D0.1cc). For the target volume, the criteria are the dose received by 1%, 95%, and 99% of the voxels within the volume (D1, D95, and D99). The DVH score is calculated for all OARs used in training (lens and retina are combined within the eye, since overlapping masks were not possible). We report the mean results for the five trained models and use one of them for a subsequent sensitivity analysis.

Additionally, the initial trained model and the updated models were tested on a set of concave target cases, a set of multiple lesion cases, and a combined test set that included both plus the standard test set.

## 3. Results

Based on the initial training set of 60 cases, the performance of the model was determined based on the standard test set of 20 cases. The mean result over five independently trained models showed a dose score, which was measured over the whole brain volume of 0.94 (standard deviation (SD) = 0.36). The mean DVH score over all OARs and the target was 1.95 (SD = 0.95).

### 3.1. Results for Sensitivity

**Figure 3:**
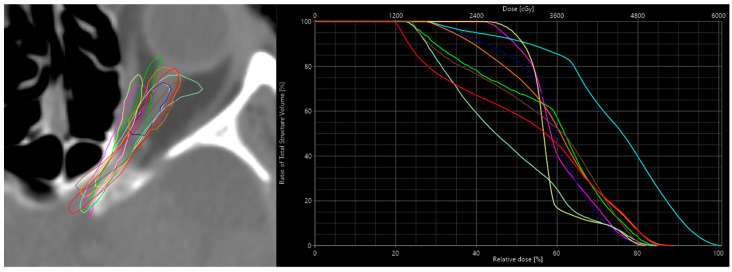
On the left is an overview of all the 9 alternative contours of the ONL. On the right, the dose’s respective DVH curves are calculated with the treatment planning system, which shows the variation in the dose these contours have. The colors in the DVH curve correspond to the colors of the contours on the left.

An overview of the nine alternative left optic nerve contours is shown in Figure 3. The mean dose to the ONL, based on the treatment planning system, and the mean dose based on the prediction model for the reference and the nine alternative contours are shown in Table 2. There is a reasonable variation in the mean dose among the different alternative contours with respect to the reference contour. In some cases, only minor changes to the mean dose occur, even though the DSC metric shows a significant difference in contour similarity. In other cases, the mean dose change with respect to the reference contour can be as high as 5 to 7 Gy. The difference between the calculated dose and the predicted dose seems to follow a trend and varies under a maximum of 3.50 Gy, with a mean of 1.38 Gy. This shows that the predicted dose is more often overestimated.

The average difference of the calculated mean dose for the alternatives with respect to the calculated reference mean dose was 2.44 Gy (i.e., the difference between the alternatives to the reference dose). For the predicted dose, this difference was 2.32 Gy. This means that the correlation coefficient between reference and predicted dose differences across the contour alternatives was 0.89, while the correlation coefficient with the DSC was −0.42 [36].

### 3.2. Improving the Model–Worst-Case Test Set

**Figure 4:**
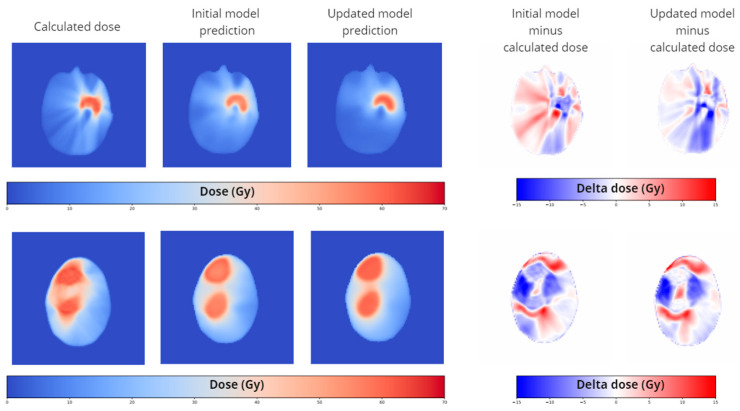
Dosimetric comparison of the calculated dose, the initial prediction model, and the updated model for a concave (**above**) and a multiple lesion case (**below**). The images represent a single axial slice. On the right, the dose difference maps of the corresponding axial slice are given. The difference between the latter shows improvements in the dose prediction. The depicted cases were not used in the training of the initial model or the updated model.

While analyzing the results of the worst-case test set, we saw flaws, particularly in cases where targets have concave shapes and consist of multiple lesions (see Figure 4). In such cases, the prediction model overestimated the ability to conform the dose to the targets. This is mainly reflected in higher dose scores and less so in the DVH scores of the target since the dose discrepancy occurs just outside of the target structure. We updated our training data with six concave target cases and six multiple lesion target cases and a combination of both. The results for the different test sets are given for the dose score, the DVH score for the OARs, and the DVH score for the targets separately in Table 3.

Based on the standard test set, the updated models scored similarly to the initial model on the dose score and the DVH score for targets. The DVH score for OARs improved for all updated models. The updated model with the concave target cases shows, by far, the best results of all trained models.

Focusing on the concave updated model, we observed improvements in the dose and DVH score on the concave test set, as well as improved results on the multiple test set. This means that, for this this small set of specific cases, the concave updated model scores significantly better than the initial model. For the multiple-lesions updated model, we also observed an improvement, but to a lesser extent. The combined updated model, containing both concave and multiple target lesions, scored the worst of all updated models. For the standard test set, the combined updated model did not show improved scores with respect to the initial model.

For the combined test set, which is a combination of the three previous test sets, the updated models scored consistently better than the initial model.

Qualitatively, we can see an improvement in the spatial distribution of the predicted dose in the updated models at exactly the locations of concern, the concave parts of the target, and the space between multiple lesions, especially in the axial direction.

## 4. Discussion

By means of an existing dose prediction model that was trained for head and neck cases, we obtained good results for translating the dose prediction model to glioblastoma cases in the brain. For these initial results, only 60 cases were used for training. Compared to the results of the OpenKBP challenge, using their proposed metrics, our initial trained model scored better values than the top ranked participants did. However, we are aware that the anatomies of the evaluated treatment sites are different. It must be noted that different 3D volumes are used, as well as different OAR structures, which will have an influence on the used scores. We do not yet have a benchmark for these metrics in the brain region. On the other hand, whereas head and neck targets are much more similar from case to case, GBM targets vary much more in size, shape, and location. Nonetheless, the model was able to achieve good results on a relatively small training set. The overall dose score was less than 1 Gy. Although this might be relevant in a final dose distribution, for the dose predictor model and the purposes we aim to use it for, we believe this to be of sufficient accuracy. Although the prediction model might have struggled in reproducing the exact streaking patterns of the actual calculated dose, this did not have an impact on the relevant metrics, such as the mean and maximum dose received by the specific OARs. We conclude from this that a cascaded 3D U-Net is capable of predicting the dose when provided with high-quality and well-curated data to train it.

Although some other groups have published on dose prediction in the brain [7,12,17], for both VMAT and conventional intensity modulated radiotherapy (IMRT), they did not specify the nature of the brain tumors. Furthermore, different treatment prescriptions were included in these works, certain tumor locations were excluded, and additional planning parameters were used in the training model. This is the first dose prediction model specified for GBM treatment.

It seems that 3D dose distribution prediction is a great application for deep-learning models. Although the predictions are not perfect, they are useful in the initialization processes of automatic planning. It also provides a near-instant estimation of the dose distribution that outperforms any currently available analytical or mathematical prediction method. Our work shows that a dose prediction model for a specific purpose, i.e., treatment area and modality, is relatively easy to obtain. For our specific purpose, we wanted to predict the dose to OARs. We were able to improve the accuracy of the DVH score for the OARs with some minor additions to the training set.

Considering it is a 3D U-Net, the training times can be relatively long. In our scaled-down version (128 by 128 by 128 voxels), a single training course lasted around 24 h. Adding more training data—for instance, doubling or tripling the amount—would be feasible. In addition, increasing the resolution to 256 voxels would improve dose prediction resolutions at the cost of longer training and inference times. On the other hand, the fast inference time of only seconds on a high-end GPU enables multiple applications where the inference is run in a high-throughput manner. Some examples where a multitude of dose predictions are used are to show the dose effect of atomic surface transformations in OARs in the brain [37] or for a clinically relevant guidance of the loss function in automatic segmentation of targets volumes for GBM [38].

Furthermore, we did not analyze the interpretability of the model. It is, however, feasible to obtain saliency maps for the outcomes of the dose prediction model since it is based on the standard U-Net architecture [39].

To obtain the training data, we used plans that were made according to a strict and standardized protocol. This makes the resulting dose distribution better to predict. It might therefore be one of the reasons for the good results. On the other hand, using such a strict protocol makes the model only valid for treatments following this strict protocol. However, in clinical practice, different approaches are used depending not only on case specifics but also on the individual preference of doctors, planners, and the availability of specific hardware. In this case, we used a VMAT technique. The dose distribution of such plans is more predictable since, in every case, two full 360-degree co-planar arcs are used. In other techniques, such as conventional IMRT, using a set number of beam angles, or in more sophisticated VMAT techniques making use of non-coplanar arcs, the dose might be more difficult to predict. To solve this issue, one needs to make a dose prediction model for every different treatment strategy. Although this seems cumbersome, in clinical reality, this comes down to a few treatment strategies per treatment site for any department. Given our model’s results, only a limited number of data are required to obtain a viable model.

The main contribution of this paper was to show the feasibility of training an accurate deep-learning-based dose predictor for GBM treatment. If data are limited for a particular scenario but a demarcated treatment protocol exists, even for non-homogeneous anatomies (i.e., not prostate or head and neck, for which most of the dose prediction methods are proposed), satisfactory results can be obtained compared to currently reported outcomes. Although the main drive behind dose prediction models is the purpose of automation in dose planning, dose prediction models can also be important for many other purposes. Our hypothesis is that they can be useful in the quality management of the radiotherapy steps that take place prior to planning. There are possible positive consequences for the effectiveness of quality assurance, the accuracy of segmentation, and the optimization of treatment planning. By introducing clinically relevant objectives early in the radiotherapy planning process, we can improve automation and enable automated quality assurance. This makes the radiotherapy process faster and also improves the quality.

We tested the obtained dose prediction model for specific criteria important for a dose predictor in quality management, sensitivity, and robustness. We showed that the initially trained model is sensitive enough to detect dose trends on realistic contour variability in a critical organ such as the optic nerve. We also tested the initial model against robustness. Although we found that the model did lack a certain accuracy in specific situations, we showed that with a simple strategy of adding specific cases to the training set, the robustness and the overall accuracy of the model increased. We anticipate that dose prediction models can be more accurate when using larger datasets of carefully curated data. In addition, the models can be tailored to have specific characteristics to fulfil the needs of different tasks in radiotherapy management.

## 5. Conclusions

This manuscript showed that obtaining a dose prediction model for GBM VMAT treatment that is both robust and sensitive to realistic segmentation variations is possible with less than 100 cases. Although the model is only valid for one specific treatment strategy, the relative ease of implementation and the near-instant inference time make it a useful technique to incorporate dose awareness into the automation processes in radiotherapy prior to planning.

## Figures and Tables

**Table 1:** Clinical dose-planning guidelines for GBM treatment.

OAR	Constraint	Priority
Brain-PTV	V_60 Gy_ ≤ 3 cc	2
Brainstem	D_0.03cc_ ≤ 60 Gy (hard constraint) D_0.03cc_ < 54 Gy	1 4
Chiasm	D_0.03cc_ ≤ 54 Gy (hard constraint) D_0.03cc_ ≤ 50 Gy	1 3
Cochlea (Ipsi-lat)	D_mean_ ≤ 45 Gy (<30% hearing loss) D_mean_ ≤ 32 Gy (<20% tinnitus)	5 9
Cochlea (Bi-lat)	D_mean_ ≤ 45 Gy (<30% hearing loss) D_mean_ ≤ 32 Gy (<20% tinnitus)	7 9
Hippocampus	D_mean_ ≤ 30 Gy (<30% IQ loss) D_0.03cc_ ≤ 30 Gy D_40%_ ≤ 7.3 Gy (long-term NCF)	8 14 11
Lacrimal Gland	D_mean_ ≤ 25 Gy (clinic) (hard constraint)	1
Lens	D_0.03cc_ ≤ 7 Gy (<25% cataract)	12
Optic nerves (Ipsi-lat)	D_0.03cc_ ≤ 54 Gy (hard constraint) D_0.03cc_ ≤ 50 Gy	1 3
Optic nerve (Bi-lat)	D_0.03cc_ ≤ 54 Gy (hard constraint) D_0.03cc_ ≤ 50 Gy	1 6
Pituitary	D_mean_ ≤ 45 Gy (panhypopituitarism) D_mean_ ≤ 20 Gy (growth hormone deficiency)	10 13
Retina	D_0.03cc_ ≤ 45 Gy (hard constraint)	1
**Target**	**Objective**	**Priority**
PTV	D_90%_ > 57 Gy (95%)	1
CTV	D_100%_ > 60 Gy (100%)	2
PTV	D_0.03cc_ < 64 Gy (107%)	3

**Table 2:** Predicted mean doses in Gy for the different optic nerve left contours.

ONL Contour	Calc. Dose	Pred. Dose	Δ Dose Calc-Pred	DSC	Δ to Calc-Ref	Δ to Pred-Ref
*Reference*	*34.7*	*35.5*	*−0.8*	*n.a.*	*n.a.*	*n.a*
Alternative-1	32.2	35.7	−3.5	0.31	−2.5	0.2
Alternative-2	30.7	32.4	−1.7	0.26	−4	−3.1
Alternative-3	34.2	34.5	−0.3	0.63	−0.5	−1
Alternative-4	31.8	34.1	−2.3	0.59	−2.9	−1.4
Alternative-5	26.9	30.1	−3.2	0.51	−7.8	−5.4
Alternative-6	32.8	36	−3.2	0.20	−1.9	0.5
Alternative-7	41.8	41.2	0.6	0.16	7.1	5.7
Alternative-8	35.3	33.1	2.2	0.58	0.6	−2.4
Alternative-9	34.5	36.1	−1.6	0.05	−0.2	0.6
**Mean**	33.49	34.87	−1.38	0.37	**Corr. Coeff.:** 0.89	

**Table 3:** Results of the dose score and DVH scores of the initial and the updated dose prediction models. Lower values represent better scores.

Test Set	Initial Model	Concave Updated Model	Multiple Lesion Updated Model	Combined Updated Model
Dose scores’ whole brain volume				
Standard test set	0.94	0.94	0.92	0.98
Concave test set	0.87	0.81	0.81	0.87
Multiple test set	1.30	0.84	1.24	1.02
Combined test set	0.98	0.90	0.95	0.97
DVH scores’ OARs				
Standard test set	2.01	1.73	1.85	1.89
Concave test set	2.11	1.67	1.99	2.08
Multiple test set	3.05	1.86	3.05	2.67
Combined test set	2.18	1.74	2.04	2.03
DVH scores’ targets				
Standard test set	1.19	1.12	1.20	1.26
Concave test set	1.72	1.67	1.51	1.66
Multiple test set	3.62	1.92	3.18	2.91
Combined test set	1.61	1.31	1.53	1.55

## Data Availability

The data presented in this study are available upon request from the corresponding author. The data on which the models are trained are not able to be shared due to privacy and ethical considerations. The data of the networks that were trained are available from third party sources, which are referred to in the main text of the manuscript.
